# *Pantoea*–*Bacillus* as a Composite Microbial Community: Inhibition and Potential Mechanism Against Potato Anthracnose Disease

**DOI:** 10.3390/jof11020121

**Published:** 2025-02-05

**Authors:** Haojie Zhang, Huiqin Shi, Mingkai Luo, Yanan Li, Wei Li, Jian Wang, Shuo Shen

**Affiliations:** 1 Academy of Agriculture and Forestry Sciences, Qinghai University, Xining 810016, China; m15713969552@163.com (H.Z.); lwbabylw@163.com (W.L.);; 2 Laboratory for Research and Utilization of Qinghai Tibet Plateau Germplasm Resource, Xining 810016, China; 3 State Key Laboratory of Plateau Ecology and Agriculture, Qinghai University, Xining 810016, China; 4 Key Laboratory of Potato Breeding of Qinghai Province, Xining 810016, China; 5 Key Laboratory of Qinghai Tibet Plateau Biotechnology, Ministry of Education, Xining 810016, China; 6 Northwest Potato Engineering Research Center, Ministry of Education, Xining 810016, China

**Keywords:** potato anthracnose, composite microbial community, antibacterial activity, defense enzymes, quantitative real-time PCR

## Abstract

The potato (*Solanum tuberosum*), an important component of global food security, often faces threats from various diseases during its growth process, especially potato anthracnose (*Colletotrichum coccodes*), which severely affects crop yield and quality. In this study, we successfully isolated and identified two bacteria with potential for biological control, (*Pantoea agglomerans*) and (*Bacillus subtilis*). The experimental results indicate that the bacterial suspensions of strains JZ-1-1-1 and JZ-2-2-2 had a significant inhibitory effect on the pathogen ZL-7, with the inhibition rate of JZ-1-1-1 reaching as high as 55.21%. The inhibition rate of JZ-2-2-2 was 53.48%. When these two strains were mixed at a 4:6 ratio, the inhibitory effect on pathogenic bacteria was even more significant, reaching 68.58% inhibition. In addition, the composite microbial community produced biofilms with their yield gradually increasing within 24 h and showing a slight decrease after 72 h. The efficacy test further indicated that the composite bacterial suspension was highly effective in controlling the spread of lesions, with an efficacy rate as high as 81.40%. In the analysis of defense enzyme activity, peroxidase (POD) and superoxide dismutase (SOD) levels peaked on day seven, while the composite bacterial suspension significantly reduced malondialdehyde (MDA) and polyphenol oxidase (PPO) activity. Quantitative real-time PCR confirmed that these two strains effectively colonized the surface of potato tubers. In summary, this study provides an important theoretical basis and practical guidance for the application of biological methods for the prevention and control of potato anthracnose.

## 1. Introduction

Potatoes (*Solanum tuberosum*) are important food crops and vegetables. This annual herbaceous plant, belonging to the Solanaceae family, possesses tubers abundant in starch, protein, vitamins, and minerals and serves as a vital energy source for humans [1,2]. Potatoes are ranked fourth among global food crops, followed by rice, wheat, and corn. Potatoes are cultivated in over 156 countries and regions, covering a total area of approximately 20 million hectares. China is one of the leading potato-producing countries, with an average annual cultivation area exceeding 5 million hectares, representing more than one-fifth of the global cultivation area [3,4,5].

The rising economic value of potatoes has resulted in expanded planting areas, exacerbating the issue of continuous cropping and facilitating the spread of detrimental bacteria. A multitude of diseases are induced by pathogens such as *Phytophthora infestans*, *Fusarium* spp., and *Colletotrichum* spp. This significantly affects the potato yield and quality. *Colletotrichum coccodes* are prevalent in over 50 nations and regions, including Japan, the United States, and the United Kingdom, and threaten many aspects of potato cultivation [6,7,8,9]. The pathogen initially causes leaf discoloration and curling in the aboveground parts of the plant. In severe cases, causing brown lesions and root rot, the entire plant may wither and die. As the disease progresses, black conidial pads and mycelia may form inside the stems of the infected plants [10,11,12,13]. The surfaces of diseased potatoes exhibit brown lesions, together with black sclerotia and toxins, which present a potential health concern to consumers [13].

In this context, the prevalent *Bacillus* in the soil effectively inhibits the growth of potato black scurf mycelia through the production of antibacterial agents, competition for nutrients, and suppression of pathogen proliferation [14]. *Pantoea agglomerans*, a Gram-negative bacterium, excels in controlling pathogenic bacteria and inhibiting wheat scab pathogens, while simultaneously reducing toxin production and enhancing crop resistance [15]. The predominant metabolites produced by *Pantoea agglomerans* include trans-cinnamic acid, antibiotics, and alkaloids, which are crucial for biological control [16]. Furthermore, *Pseudomonas* GZ-33 can be used to effectively manage bacterial wilt by treating the soil with a bacterial liquid or spraying it onto the crop’s surface [17]. Antimicrobial agents not only eliminate infections directly but also mitigate their detrimental effects on plants by suppressing plant growth and reproduction [18].

Currently, biological control methods predominantly rely on the isolation and application of individual strains. However, in practical agricultural production, biocontrol agents with a singular component frequently encounter issues such as inconsistent efficacy and a restricted inhibition range [19]. Research indicates that the combined application of biocontrol microbial communities exhibiting no mutual antagonism can effectively address the limitations of individual microbial agents, including their ambiguous field effects, restricted antibacterial spectra, and the inability to fully harness their potential. To address these challenges, a strategy involving the combination of multiple distantly related strains that do not exhibit antagonism, was implemented and applied concurrently in the field. This enables these strains to perform their biological control roles, collaborate, and collectively combat plant diseases, thereby effectively increasing the control efficacy and substantially boosting the ecological structure of the soil [20,21]. Composite microbial communities exhibit diversity and are generally composed of many types of bacteria, fungi, and actinomycetes [22]. Researchers devise combinations based on modes of action, application objectives, environmental adaptability, and the attributes of biocontrol strains. In practical use, various microorganisms with distinct functions that can coexist and provide mutual benefits are often selected, combined in specific ratios, and adsorbed through a particular medium to form fertilizers with defined features [23,24]. Consequently, composite microbial communities not only enhance soil microbial populations, but also efficiently suppress pathogen proliferation, thereby facilitating effective pest and disease management in crops [25]. Potato anthracnose scurf is a significant disease encountered during potato cultivation. Infection by black scurf fungus induces withering of potato plant leaves, impairs normal growth and development, and ultimately results in significant yield and quality losses. There are numerous reports on the use of single strains to mitigate fungal illnesses in plants.

This study investigated the potential of composite microbial communities to prevent and control potato anthracnose through the isolation, identification, and antibacterial activity testing of microorganisms and the analysis of their effects on potato defense mechanisms. This study offers a theoretical foundation and practical assistance for managing plant diseases, while also presenting a novel perspective on mitigating the low biocontrol efficacy of individual strains.

## 2. Materials and Methods

### 2.1. Isolation and Cultivation of Microorganisms

*Pantoea agglomerans* JZ1-1-1 and *Bacillus subtilis* JZ2-2-2 were isolated and purified from potato variety 10. The test pathogen is *Colletotrichum coccodes* (ZL-7), which was isolated from potato sample No. 10. The potato sample was ground using a sterile mortar and pestle, and the supernatant was repeatedly diluted. The resulting supernatant was spread on agar plates for cultivation, and the isolated strain was continuously purified for 2–3 generations [26,27]. The experiment involved two types of media: PDA medium, which contained 200 g of peeled potatoes, 20 g of glucose, 20 g of agar powder, and 1000 mL of distilled water, maintaining a natural pH value [26]; and LB liquid medium, composed of 10 g of NaCl, 10 g of peptone, 5 g of yeast extract, and 1000 mL of distilled water, maintaining a natural pH. The LB solid medium was prepared by adding 20 g of agar powder to the above [27]. All aforementioned strains were preserved in the Microbiology Laboratory of the Qinghai Academy of Agricultural and Forestry Sciences.

### 2.2. Identification of Microorganisms

Using a bacterial genomic DNA extraction kit (Bioray Biotechnology Co., Ltd., Beijing, China), we extracted the genomic DNA of strains JZ-1-1-1 and JZ-2-2-2. For the identification of *Bacillus* species, conserved genes such as 16S rDNA, *gyrA*, *atpD*, and *rpoB* were targeted; for the identification of *Paenibacillus* species, conserved genes such as 16S rDNA, *gyrB*, *frr*, and *rpoD* were targeted. PCR amplification and sequencing were performed (primer sequences are detailed in Table 1 [28,29,30,31]. After detection and recovery of the PCR products by 1% agarose gel electrophoresis, the products were sent to Aiyouji Biotechnology Co., Ltd. (Xi’an, China). for sequencing. Using MEGA 7.0 and Sequence Matrix software version 1.7.8, the above gene sequences were assembled and aligned, and a phylogenetic tree was constructed using the maximum likelihood method (with a bootstrap value of 1000) to determine the taxonomic status of strains JZ-1-1-1 and JZ-2-2-2. After passing the electrophoresis test, sequencing was performed, and the sequencing results were submitted to the NCBI GenBank database for homologous comparison, followed by the construction of a phylogenetic tree [32].

### 2.3. Construction of the Composite Microbial Community

Strain seed liquid was prepared by selecting single colonies from JZ-1-1-1 and JZ-2-2-2, inoculating them into the LB liquid medium, and incubating overnight at 28 °C with shaking at 180 rpm. Fermentation broth was prepared by transferring the seed culture at a 1% inoculation rate to an LB liquid medium and incubating under the same conditions for 72 h. Sterile fermentation broth was prepared by centrifuging at 12,000 rpm for 15 min at 4 °C, collecting the supernatant and removing the cells using a 0.22 µm filter membrane. The bacterial suspension was prepared. The cells were centrifuged, washed with PBS, and resuspended in the original volume [33,34]. Seed cultures of JZ-1-1-1 and JZ-2-2-2 were inoculated into LB liquid medium at ratios of 1:9, 2:8, 3:7, 4:6, 5:5, 6:4, 7:3, 8:2, and 9:1, with a total inoculation volume of 1%, to prepare the cell suspension and fermentation broth [33,34].

### 2.4. Determination of Antimicrobial Activity of Composite Microbial Communities

The antimicrobial activities of the fermentation broth and microbial suspensions were determined using the filter paper disc method. The antimicrobial rate was calculated by measuring the diameters of colonies in the control and treatment groups using the Cross method [35].

Inhibition rate (%) = [(Diameter of control pathogen − Diameter of treated pathogen)/Diameter of control pathogen] × 100(1)

### 2.5. Determination of Biofilm Formation Ability of Mixed Microbial Communities

The procedure for forming biofilms with the composite microbial community was conducted as previously outlined [36]. Strains JZ1-1-1 and JZ2-2-2 were inoculated into 100 mL of LB liquid medium in a 4:6 ratio and incubated overnight at 28 °C. The cells were then collected, and their density was adjusted to 1 × 10^7^ cells mL^−1^. Aliquots of 1500 μL were added to a 24-well plate containing 1000 μL of LB medium, with an equal volume of LB medium used as a control. Samples were harvested at 0, 48, and 96 h to assess the biofilm formation ability of the composite microbial community using the crystal violet method [36]. The absorbance of the acetic acid solution was measured at OD_590_ to evaluate biofilm formation. Each treatment included three replicates, and the experiment was repeated three times.

### 2.6. Control Effect of Composite Microbial Communities on Potato Anthracnose and Their Impact on Defense Enzyme Activity

Healthy, undamaged potato tubers (120–150 g) were washed with sterile water, soaked in 1% sodium hypochlorite solution for 3 min, and rinsed 3–5 times with sterile water before being air-dried. A sterile punch was used to create a hole (0.8 cm × 1.5 cm) in the middle of the tuber. The experimental design consisted of six groups, with 25 samples per group, and was repeated thrice. The control group (S) used sterile water, 80 mL per well, the pathogen-only group (T) was inoculated with anthrax spore suspension (1 × 10^6^ spores/mL), 80 mL per well. The prevention group (Y) was first inoculated with 40 μL of composite bacterial suspension (OD_600_ = 1.0) and then inoculated with 40 μL of anthrax spore suspension after 24 h. (X) The treatment group was only inoculated with the mixed microbial suspension (1 × 10^6^ spores/mL), adding 80 μL per well. (M) In the potato tuber hole, first inoculate with 40 μL of prochloraz (recommended drug concentration), and then inoculate with 40 μL of spore anthrax suspension after 24 h. (Z) was first inoculated with 40 μL of anthrax spore suspension, and then inoculated with 40 μL of the composite bacterial suspension after 24 h. The treated potatoes were sealed in sterile bags and stored at 28 °C with humidity. Samples were collected on days 1, 3, 5, 7, 9, and 11 to observe the occurrence of potato dry rot, and the weight loss rate and control efficacy were calculated on day 11 [37,38]. The calculation formulae are as follows:

Weight loss rate/% = [(Weight of tuber on day 11 − Weight of tuber on day 0)/Weight of tuber on day 11] × 100%(2)

Control efficacy/% = [(Diameter of the lesion in the control group − Diameter of the lesion in the treatment group)/(Diameter of the lesion in the control group − Diameter of puncture)] × 100%(3)

Samples were collected on days 1, 3, 5, 7, 9, and 11 after the treatment to evaluate the effects of the composite microbial community on the activity and composition of potato defense enzymes. We measured the concentration of malondialdehyde (MDA), and polyphenol oxidase (PPO) activity was referenced from [39]. The activity assays for superoxide dismutase (SOD) and peroxidase (POD) were conducted according to [40].

### 2.7. Specific Design of Primers and Probes

Based on the differences in the 16S rDNA gene sequences of strains JZ-1-1-1 and JZ-2-2-2 and their closely related species, specific primers and probes were designed using Primer Express 3.0 software [41,42]. The designed primers include BF-(5′-ACGATGAGTGCTAAGTGTTA-3′), BR-(5′-AGGATTGTCAGAGGATGTC-3′), PF-(5′-GGCTAATACCGCATAACG-3′), and PR-(5′-GTGTCTCAGTTCCAGTGT-3′). PCR amplification was performed using these specific primers under the following conditions: denaturation at 98 °C for 10 s, annealing at 60 °C for 30 s, and extension at 72 °C for 30 s, for a total of 30 cycles. The amplification products were detected using 2% agarose gel electrophoresis. The primers and probes were synthesized by Sangon Biotech Co., Ltd. (Shanghai, China).

### 2.8. Real-Time Quantitative PCR Analysis of the Colonization Ability of Complex Microbial Communities on Potato Tubers

After amplifying a specific fragment of the target strain using specific primers, it was inserted into a pMD19-T vector. After linearization with Pst I restriction endonuclease, a series of gradient dilutions were performed to obtain DNA dilutions of 10^1^ to 10^7^ copies/μL [43,44]. Using a series of dilutions as templates, quantitative real-time PCR amplification was performed under the conditions described in Section 2.7. A standard curve was established using the logarithm of the plasmid copy number as the x-axis and the cycle threshold (Ct) of qPCR as the y-axis. Next, a series of dilutions were used as templates for quantitative fluorescence PCR amplification. The PCR reaction system was 20 μL, consisting of: 10 μL Premix Ex Taq, 0.4 μL upstream primer LHHF-F (10 μM), 0.4 μL downstream primer LHHF-R (10 μM), 0.8 μL probe LHHF-P (10 μM), and 2 μL DNA template, with RNase-free ddH_2_O added to a final volume of 20 μL. The reaction was conducted on the Roche Diagnostics LightCycler^®^/LightCycler^®^ 480 instrument (Roche Diagnostics, Tokyo, Japan), with the specific reaction program as follows: denaturation stage at 95 °C for 30 s (1 cycle), PCR cycling stage at 95 °C for 5 s and 60 °C for 30 s (a total of 40 cycles), and cooling stage at 50 °C for 30 s (1 cycle). The mixed bacterial suspension (OD_600_ = 1.0) was injected into the potato tubers, and a plant DNA kit was used to extract DNA from the potato tubers on days 1, 7, and 11. (The kit was provided by Biorise Biotechnology Co., Ltd. (Beijing, China)).

### 2.9. Statistics and Analysis

Each treatment group consisted of three samples; each experiment was conducted at least three times. Data were statistically analyzed using Excel (2019) and SPSS 25.0, and significance analysis was performed using a one-way ANOVA (*p* < 0.05). Graphs were plotted using Origin9 software 2024 version (OriginLab Corporation, Northampton, MA, USA).

## 3. Results

### 3.1. Identification of Biocontrol Bacteria

**Figure 1:**
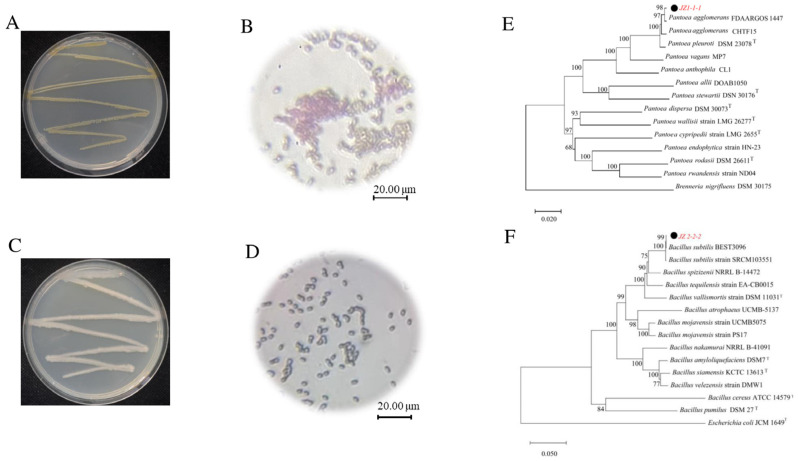
Morphological characteristics and phylogenetic tree of different bacteria. (**A**) Morphological characteristics of JZ1-1-1 on LB medium; (**B**) Micromorphology of JZ1-1-1 cells, scale bar = 20 μm; (**C**) Morphological characteristics of JZ2-2-2 on LB medium; (**D**) Micromorphology of JZ2-2-2 cells, scale bar = 20 μm; (**E**): Phylogenetic tree of JZ1-1-1 constructed based on the sequences of 16S rDNA, *gyrB*, *frr*, and *rpoD* genes. This tree was constructed using the neighbor-joining method, and there 1000 lead repetitions show a lead rate greater than 50%. (**F**) Phylogenetic tree of JZ2-2-2 constructed based on the sequences of 16S rDNA, *atpD*, *gyrA*, and *rpoB* genes. This tree was constructed using the neighbor-joining method, and there 1000 lead repetitions show a lead rate greater than 50%.

Strain JZ-1-1-1, isolated from potato 10, forms yellow colonies, has rod-shaped cells, is a Gram-negative bacterium that can produce yellow pigments and has smooth, well-defined colony edges (Figure 1A,B). Preliminary morphological examination identified the bacterium *Pantoea* sp. To further clarify the taxonomic status of the biocontrol bacterium, a multigene phylogenetic tree of strain JZ-1-1-1 was constructed based on its 16S rDNA, *gyrB*, *frr*, and *rpoD* gene sequences of strain JZ-1-1-1 (Figure 1E). These results indicated that strain JZ1-1-1 clustered with *Pantoea agglomerans* FDAARGOS1447 in the same branch. Strain JZ-1-1-1 was identified as *Pantoea agglomerans*.

Strain JZ-2-2-2, isolated from potato 10, formed white colonies with cells appearing as straight rods, is a Gram-positive bacterium that can produce a uniformly stained, rough, opaque spread (Figure 1C,D). Morphological examination preliminarily identified the bacterium as belonging to *Bacillus* sp. To further clarify the taxonomic status of the biocontrol bacteria, a multigene phylogenetic tree of strain JZ-1-1-1 was constructed based on the gene sequences of 16S rDNA, *atpD*, *gyrA*, and *rpoB* (Figure 1F). The results indicated that strain JZ2-2-2 clustered with *Bacillus subtilis* BEST3096 in the same branch, and strain JZ-1-1-1 was identified as *Bacillus subtilis*.

### 3.2. Antimicrobial Activity of the Composite Microbial Community Against Potato Anthracnose

**Figure 2:**
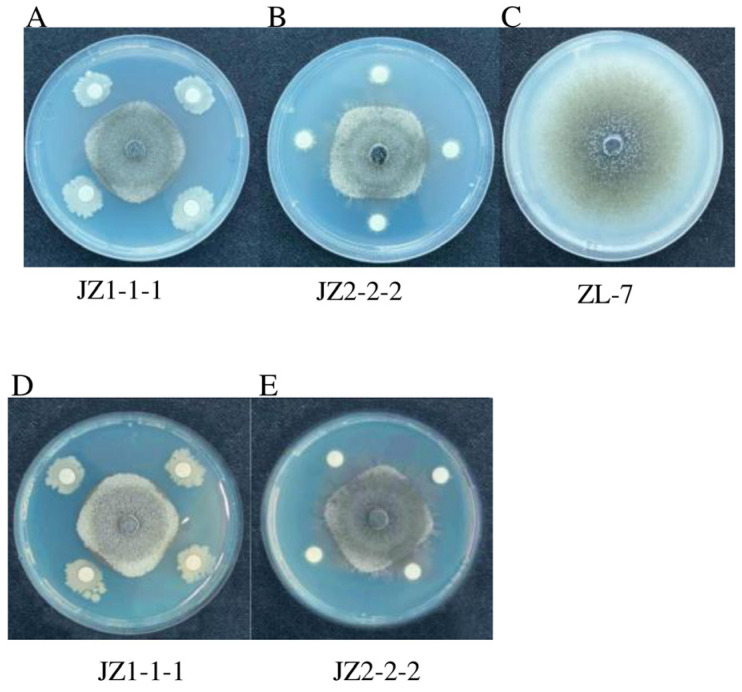
Antimicrobial activity of different fermentation products against *Colletotrichum coccodes*. (**A**) Antimicrobial activity of JZ1-1-1 bacterial suspension against *Colletotrichum coccodes*; (**B**) Antimicrobial activity of JZ2-2-2 bacterial suspension against *Colletotrichum coccodes*; (**C**) *Colletotrichum coccodes* ZL-7; (**D**) Antimicrobial activity of JZ1-1-1 fermentation broth against *Colletotrichum coccodes*; (**E**) Antimicrobial activity of JZ2-2-2 fermentation broth against *Colletotrichum coccodes*.

**Figure 3:**
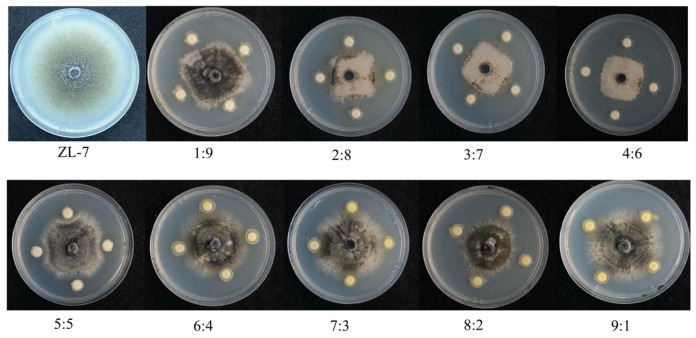
Antifungal activity of different ratios of JZ1-1-1 and JZ2-2-2 composite bacterial suspensions against *Colletotrichum coccodes*.

To further investigate the antimicrobial activity of the selected composite microbial communities against potato anthracnose, antimicrobial experiments were conducted on the fermentation products of the two strains, as shown in Figure 2A,B. The fermentation liquids of strains JZ1-1-1 and JZ-2-2-2 both exhibited certain antimicrobial activity against the pathogen ZL-7, with strain JZ1-1-1 showing an inhibition rate of 51.46%, which was higher than that of strain JZ-2-2-2, which had an inhibition rate of 48.29%. Similarly, the bacterial suspension of strain JZ1-1-1 exhibited a higher antibacterial effect against the pathogen ZL-7 compared to the bacterial suspension of strain JZ-2-2-2, reaching 55.21%, as shown in Table 2 as well as in Figure 2D,E. Therefore, the effects of the bacterial suspension were superior to those of the fermentation broth. Therefore, different ratios of the bacterial suspensions of strains JZ-1-1-1 and JZ-2-2-2 were used, as shown in Figure 3. At a 4:6 ratio of strains JZ-1-1-1 and JZ-2-2-2, the antibacterial effect against the pathogen ZL-7 was the highest, achieving an antibacterial rate of 68.58%, as shown in Table 3. Therefore, subsequent experiments were performed using this ratio.

### 3.3. Biofilm Formation Ability of the Composite Microbial Community

**Figure 4:**
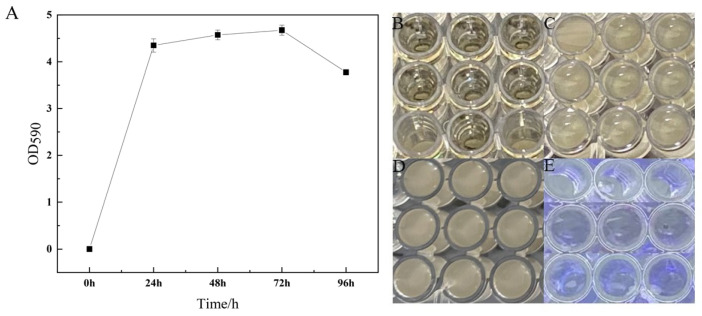
Biofilm formation ability of the composite microbial community at 28 °C. (**A**) Biofilm morphology of the composite microbial community at 0–96 h (**B**) 0 h (**C**) 48 h (**D**) 96 h. (**E**) 0, 48, and 96-h states of crystal violet-stained biofilms at different time intervals.

The composite microbial community demonstrated the ability to form biofilms (Figure 4A). Biofilm yield increased steadily from 0 to 24 h, stabilized between 24 and 72 h, and then declined after 72 h. There was no significant difference in biofilm formation between 24 and 72 h; however, during the 12 to 24-h period, the biofilm was relatively loose and susceptible to detachment. From 24 to 72 h, the structure of the biofilm remained intact, while dissociation occurred from 72 to 96 h, with some cells appearing in a planktonic state (Figure 4B–D). Following crystal violet staining, a purple ring was observed on the inner wall of the well plate, confirming the biofilm-forming capability of the composite microbial community. Nevertheless, the intensity of the purple ring gradually diminished over time, indicating a potential weakening of the community’s biofilm-forming ability (Figure 4E).

### 3.4. Determination of the Efficacy of Mixed Microbial Communities Against Potato Tuber Anthracnose

**Figure 5:**
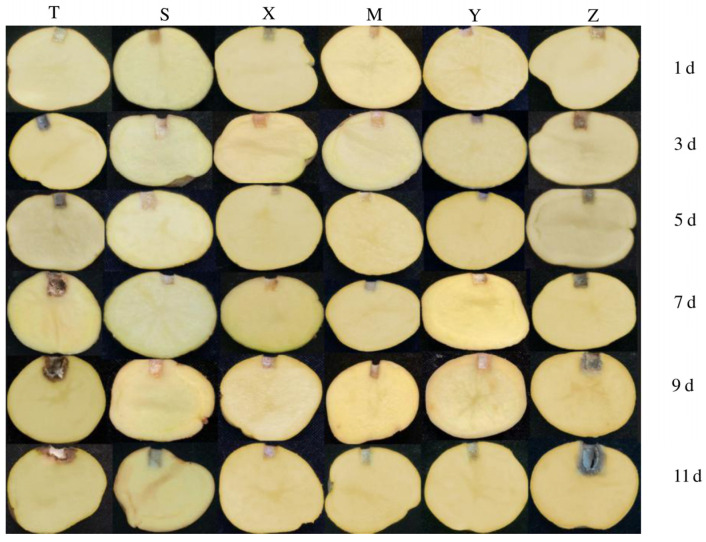
The condition of potato tubers at different growth stages. Note: (T): Potato Pathogen ZL-7, (S): Control, (X): Composite Microbial Community, (M): Prochloraz Mode, (Y): Preventive Mode, (Z): Therapeutic Mode.

As shown in Figure 5 and Table 4, with an increase in storage time, the lesions gradually expanded. In the pathogen treatment group (T), the lesions expanded rapidly, especially on the 11th day, when the interior of the potatoes became rotten and hollow, with significant moisture loss and the highest weight loss rate of 22.58%. The treatment group (Z) shows similar changes on the 11th day, with hollowness inside the potatoes and a weight loss rate of 15.45%, resulting in a prevention efficacy of 44.47%. In contrast, the preventive model treatment group (Y) and prochloraz treatment group (M) exhibited relatively slower changes in lesion development and diameter than the treatment group. This result was consistent with the prevention efficacy and weight loss rates. Therefore, it can be concluded that during storage, the suspension of the composite microbial community can inhibit the weight loss of stored potatoes caused by the pathogen ZL-7, thereby maintaining the weight of the tubers.

### 3.5. The Effect of the Composite Microbial Community on Defense Enzyme Activity Against Potato Anthracnose Disease

**Figure 6:**
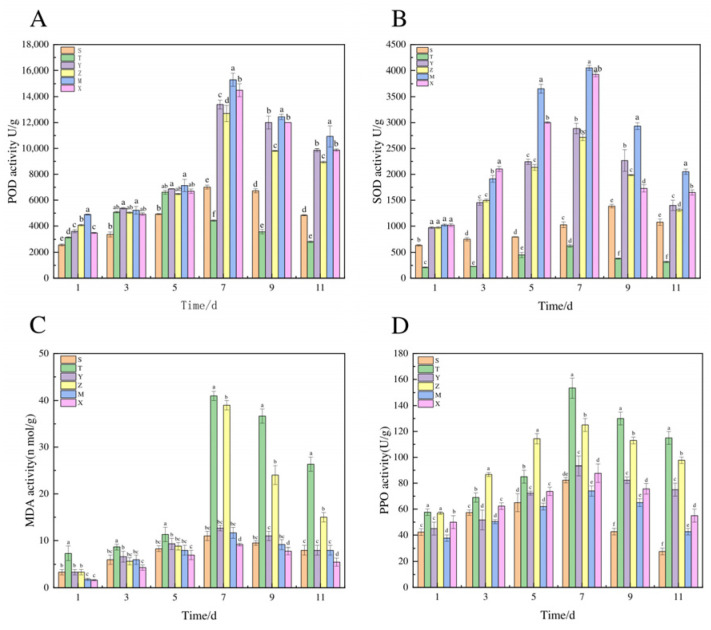
The effect of the composite microbial community on potato-related defense enzymes. Note: (**A**) POD; (**B**) SOD; (**C**) MDA; (**D**) PPO. Note: (T): Potato Pathogen ZL-7, (S): Control, (X): Composite Microbial Community, (M): Pro-chloraz Mode, (Y): Preventive Mode, (Z): Therapeutic Mode. Different lowercase letters represent the differences between different treatments (*p* < 0.05).

As shown in Figure 6A,B, POD and SOD activities in each treatment initially increased and then decreased, peaking on the 7th day. The enzyme activities, from high to low, were as follows: Prochloraz Mode (M) > Composite Microbial Community (X) > Preventive Mode (Y) > Therapeutic Mode (Z) > Control (S) > Pathogen ZL-7 (T). The Preventive Mode (Y) exhibited good enzyme activity in all treatments, which was significantly higher than that in the Therapeutic Mode (Z), Control (S), and Pathogen (T), indicating that preventive measures can be effective. Therefore, although the effects of mefenoxam and the composite microbial community were relatively good, the preventive model remains an effective strategy for enhancing plant health and resistance.

From Figure 6C, it can be observed that the changes in potato MDA enzyme activity in the different treatment groups show a trend of first rising and then falling. In contrast, the pathogen control group (T) shows a trend of first increasing and then decreasing, reaching a maximum value of 41.0 nmol/g on the 7th day. The composite microbial control group (X), preventive model group (Y), and therapeutic model (Z) were all significantly lower than the pathogen control group (T). The composite microbial community significantly enhanced the potato defense enzyme activity.

In terms of PPO enzyme activity, compared with the T group, the changes were most significant, showing varying degrees of reduction in the treatments with the inoculated microbial community, and all exhibited an initial increasing trend followed by a decrease. The preventive treatment group, which involved inoculating the composite microbial community for 24 h before inoculating the pathogenic bacterium ZL-7, exhibited significantly reduced PPO enzyme activity compared to the therapeutic treatment group, as shown in Figure 6D.

### 3.6. Specificity Verification of Primers and Probes

**Figure 7:**
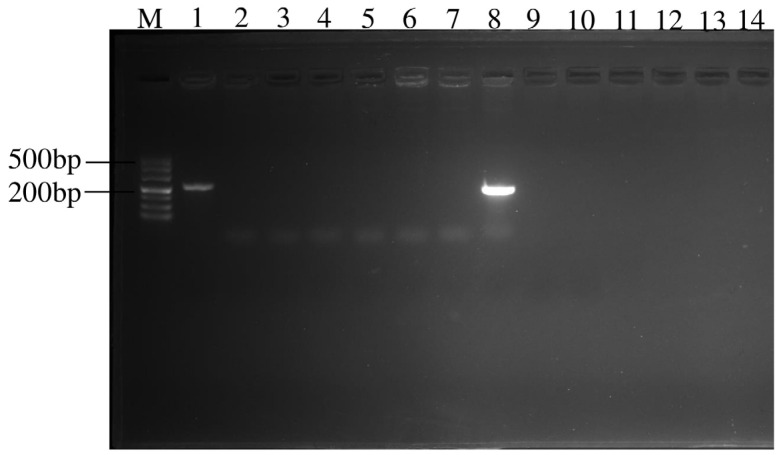
Electrophoresis results of related primers amplification for strains JZ1-1-1 and JZ2-2-2. Note: M: DNA Marker 500; 1: JZ1-1-1; 2: JZ2-2-2; 3: *Bacillus amyloliq-uefaciens* QS10-6; 4: *Bacillus atrophaeus* QS2-5; 5: *Bacillus velezensis* QS2-13; 6: *Bacillus safensis* BM-7; 7: *Bacillus atrophaeus* Q2-7; 8: JZ2-2-2; 9: JZ1-1-1; 10: *Bacillus amyloliq-uefaciens* QS10-6; 11: *Bacillus atrophaeus* QS2-5; 12: *Bacillus velezensis* QS2-13; 13: *Bacillus safensis* BM-7; 14: *Bacillus atrophaeus* QB-2-7.

Using the specific primers strains JZ-1-1-1 and JZ-2-2-2, quantitative PCR detection was performed on the genomic DNA of the five *Bacillus* strains. The amplification effect of the primers was specific, with no non-specific bands appearing, making them suitable as specific primers for quantitative PCR detection, as shown in Figure 7. As shown in Table 5, the results indicate that using the genomic DNA of the strain JZ-1-1-1 as a template yielded a strong amplification signal (Ct = 29.85), whereas using the genomic DNA of the other five *Bacillus* strains as templates did not yield an amplification signal. Using the genomic DNA of strain JZ-2-2-2 as a template yielded a strong amplification signal (Ct value = 28.74), whereas the genomic DNA of the other five *Bacillus* strains did not produce an amplification signal. Using potato tuber DNA as a template did not yield an amplification signal, but adding the same concentration of strain JZ-1-1-1 genomic DNA to potato tuber genomic DNA produced a strong amplification signal (Ct value = 26.60). Similarly, using potato tuber DNA as a template did not yield an amplification signal, but adding the same concentration of strain JZ-2-2-2 genomic DNA to potato tuber genomic DNA produced a strong amplification signal (Ct = 25.72), as shown in Table 5. These results demonstrate that the primers and probes designed in this study for strains JZ-1-1-1 and JZ-2-2-2 have high specificity.

### 3.7. Fluorescent Quantitative PCR Analysis of the Colonization Ability of Composite Microbial Communities on Potato Tubers

**Figure 8:**
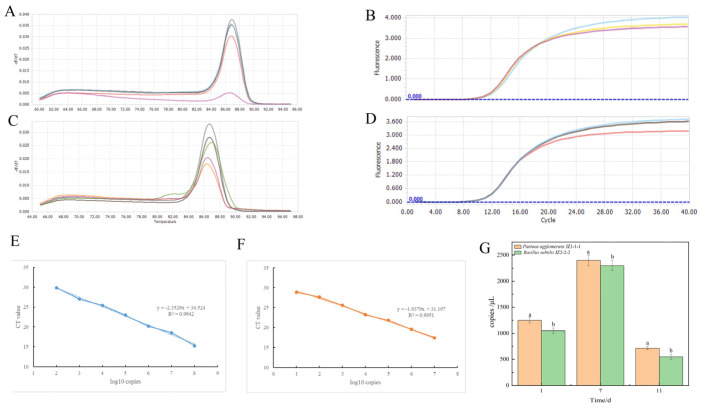
Fluorescent quantification and colonization detection of different strains. (**A**) PCR Dissociation Curve of Strain JZ1-1-1.The curves of different colors represent the dissolution curve of strain JZ1-1-1. (**B**) Amplification Curve of Strain JZ1-1-1, the curves of different colors represent the amplification curve of strain JZ1-1-1. (**C**) PCR Dissociation Curve of Strain JZ2-2-2 The curves of different colors represent the dissolution curve of strain JZ2-2-2. (**D**) Amplification Curve of Strain JZ2-2-2, the curves of different colors represent the amplification curve of strain JZ2-2-2 (**E**) Curve Equation Established by Ct Value (Y) and DNA Concentration (X) of Strain JZ1-1-1, the dotted line represents the trend line of strain JZ1-1-1. (**F**) Curve Equation Established by Ct Value (Y) and DNA Concentration (X) of Strain JZ2-2-2, the dotted line represents the trend line of strain JZ2-2-2. (**G**) Detection of colonization levels of strain JZ 1-1-1 and strain JZ 2-2-2 in potato tubers. Different lowercase letters represent the differences between different treatments (*p* < 0.05).

As shown in Figure 8A,B, real-time fluorescent quantitative PCR detection of strain JZ-1-1-1 revealed a single peak in the melting curve, indicating high accuracy. According to the Ct value (Y) of quantitative PCR and DNA concentration (X), the fitted curve equation (7C) is: y = −2.3529x + 34.524, R^2^ = 0.9942. From Figure 8D,E, it can be seen that the melting curve and amplification curve peak shape of strain JZ-2-2-2 in real-time quantitative PCR detection analysis are single and highly accurate. According to the Ct value (Y) and DNA concentration (X) of quantitative PCR, the fitted curve equation (7F): y = −1.9379x + 31.197, R^2^ = 0.9951. Strains JZ-1-1-1 and JZ-2-2-2 can colonize potato tubers, and the colonization quantity shows a decreasing trend over time, with the highest colonization amounts detected in the 7-day samples, being 2400 and 2300 copies/μL, respectively, as shown in Figure 8G.

## 4. Discussion

In the present study on biocontrol bacteria identification, we successfully isolated two strains, JZ-1-1-1 and JZ-2-2-2, identified as *Pantoea agglomerans* and *Bacillus subtilis*, respectively. The potential applications of these two bacteria in agriculture are noteworthy. *Pantoea* bacteria have been shown to inhibit various plant pathogens, demonstrating good biocontrol effects [45], while *Bacillus* bacteria are renowned for producing a variety of antimicrobial metabolites and are widely used in the prevention and control of plant diseases [46].

In the antibacterial experiment, the fermentation broths of JZ-1-1-1 and JZ-2-2-2 both exhibited significant inhibitory effects on the potato anthracnose ZL-7, with JZ-1-1-1 showing a higher inhibition rate of 51.46%, indicating that this strain may possess stronger biocontrol capabilities. The antibacterial effect of the bacterial suspension was observed to be better than that of the fermentation broth, indicating that the viable cells of the bacteria and their metabolic products play a key role in inhibiting the growth of the pathogen. These live cells can effectively inhibit the growth of pathogenic bacteria by producing antibacterial substances, competing for nutrients, and occupying space. Previous studies indicate that live cells are significantly more effective than dead cells or culture media containing only metabolic products in inhibiting pathogens [47,48].

The biofilm formation of composite microbial communities is of great significance in the prevention and control of potato anthracnose. Potato anthracnose is a severe disease caused by the fungus *Colletotrichum* spp., which significantly affects the yield and quality of potatoes. The composite microbial community can effectively enhance the plant’s disease resistance and inhibit the growth of pathogens through the formation of its biofilm [48]. In addition, biofilms can also colonize within the plant, providing protection and preventing pathogen infection [49]. Research has found that when the biofilm formation ability of *Bacillus amyloliquefaciens* HY96-2 weakens, its effectiveness in controlling tomato bacterial wilt significantly decreases [50]. The results of this study show that the biofilm formation ability of this strain gradually increased from 0 to 24 h and stabilized from 24 to 72 h. This phenomenon may be related to the synergistic effects among the constituent strains in the composite microbial community, which can produce antimicrobial substances such as antibiotics and volatile organic compounds. These substances can directly inhibit the growth of anthrax pathogens [51]. As time progresses, the ability to form biofilms decreases, which is related to the metabolic waste produced by bacteria during their growth. These substances may inhibit bacterial growth and the stability of biofilms. The accumulation of metabolic products may lead to changes in the physiological state of bacteria, thereby affecting the formation of their biofilms, as is consistent with the research [52].

In the efficacy assessment of potato anthracnose, our results showed that the composite microbial community’s preventive mode was significantly more effective than the therapeutic mode [53]. This finding indicates that preventive measures are more effective in disease management, which is consistent with the results of other related studies [54]. Biocontrol bacteria have shown significant potential for preventing plant diseases and effectively inhibiting infection by pathogenic microorganisms [55].

In terms of defense enzyme activity, our study found that the activities of POD and SOD peaked on the 7th day after treatment, indicating that inoculation with biocontrol bacteria significantly stimulated the defense response in potatoes. Enzyme activity was significantly higher in the preventive mode group than in the other groups, indicating that biocontrol bacteria can effectively induce the plant’s endogenous defense mechanisms. This result is consistent with studies showing that biocontrol bacteria enhance plant resistance to pathogens by inducing defense enzymes in plants [56,57].

The present study showed that the inoculation of composite microbial communities significantly increased defense enzyme activity in potatoes, particularly in the preventive mode, where the plant resistance to stress was notably enhanced. This further supports the effectiveness of early inoculation with biocontrol bacteria, which can enhance the resistance of plants to pathogens, thereby effectively reducing the incidence of diseases. Related studies have shown that biocontrol bacteria enhance the overall disease resistance of plants by activating the plant disease-resistance genes and promoting the synthesis of enzymes involved in the defense mechanism [58].

This study validated the effectiveness of specific primers and probes for strains JZ-1-1-1 and JZ-2-2-2 in quantitative PCR. The results showed that the designed primers had high specificity and sensitivity, effectively distinguishing the target strains from five other *Bacillus* strains. In the experiment, only JZ-1-1-1 and JZ-2-2-2 obtained significant amplification signals, while other strains did not amplify, indicating that the primer design was successful and effectively prevented non-specific amplification. When using the JZ-1-1-1 and JZ-2-2-2 DNA templates, the Ct values were 29.85 and 28.74, respectively, indicating good amplification capability. When the target strain DNA was mixed with potato tuber DNA, the Ct values were 26.60 and 25.72, respectively, further confirming the specificity and sensitivity of the primers. Fluorescent quantitative PCR analysis showed that the colonization abilities of JZ-1-1-1 and JZ-2-2-2 in potato tubers were 2400 and 2300 copies/μL, respectively, indicating that the composite microbial community could successfully colonize, and the colonization numbers gradually decreased over time. This phenomenon may be caused by environmental factors, nutrient depletion, or a plant defense response [59].

## 5. Conclusions

In this study, we successfully isolated and identified two biocontrol bacteria, *Pantoea agglomerans* and *Bacillus subtilis*, and explored the potential of the combined microbial community in controlling potato anthracnose. The results indicated that the composite microbial community exhibited significant antibacterial activity against pathogens, formed biofilms, and enhanced the activity of defense enzymes in potatoes. Additionally, it can effectively colonize potato tubers, particularly under preventive inoculation conditions, thereby significantly improving plant resistance to stress. These findings provide a theoretical basis and practical guidance for the biological control of potato anthracnose, and highlight the advantages of integrating biocontrol bacteria into disease management. Future research should focus on optimizing the ratio of strains, clarifying their mechanisms of action, and evaluating their effectiveness in practical production to provide new solutions for disease management in potatoes and other crops.

## Figures and Tables

**Table 1:** Primer information for molecular identification.

Gene	Primer	Primer Sequences
16sDNA	F	AGAGTTTGATCCTGGCTCAG
	R	GGTTACCTTGTTACGACTT
gyrA	gyrA-F	CAGTCAGGAAATGCGTACGTCCTT
	gyrA-R	CAAGGTAATGCTCCAGGCATTGCT
atpD	atpD-F	ACAAGTTTGTCTTCCTCGCC
	atpD-R	TGAGGTCGCTCTTCTTTAGG
rpoB	rpoB-F	GGAAACCGCCGTTTTACGTTC
	rpoB-R	CCATGAGGCACACGAAGAGA
gyrB	gyrB-F	ATTGGTGACACCGATCAAACA
	gyrB-R	TCATACGTATGGATGTTATTC
frr	frr-F	AAACCACCATCAGCAAAG
	frr-R	TTGGGTTCAGACCGAGAT
rpoD	rpoD-F	TAGCCGAATACCCTGAAGC
	rpoD-R	AAATCGCCCAGATGTGAA
ITS	F	GGTTTTGATCCTTGTCTCCAG
	R	GGTTACCTGTTACGACTT

**Table 2:** Antimicrobial activity of different fermentation products against *Colletotrichum coccodes*.

	Fermentation Broth		Bacterial Suspension	
Strain	Colony Diameter (cm)	Inhibitory Rate (%)	Colony Diameter (cm)	Inhibitory Rate (%)
ZL-7	7.01 ± 0.12 a	-	-	-
JZ1-1-1	3.43 ± 0.08 b	51.46	3.17 ± 0.05 c	55.21
JZ2-2-2	3.61 ± 0.07 b	48.9	3.29 ± 0.06 c	53.48

Note: The above values represent the mean ± standard error. Different lowercase letters in the same column indicate significant differences between groups of the same fermentation products treated with different strains (*p* < 0.05). “-” indicates no inhibitory activity.

**Table 3:** The antibacterial activity of different ratios of JZ1-1-1 and JZ2-2-2 bacterial suspensions against *Colletotrichum coccodes*.

	Bacterial Suspension	
Strain	Colony Diameter (cm)	Inhibitory Rate (%)
ZL-7	7.01 ± 0.12 a	-
JZ1-1-1 and JZ2-2-2 (1:9)	4.05 ± 0.09 c	42.65
JZ1-1-1 and JZ2-2-2 (2:8)	3.56 ± 0.05 d	49.71
JZ1-1-1 and JZ2-2-2 (3:7)	3.21 ± 0.04 e	54.75
JZ1-1-1 and JZ2-2-2 (4:6)	2.25 ± 0.03 f	68.58
JZ1-1-1 and JZ2-2-2 (5:5)	5.52 ± 0.10 b	21.46
JZ1-1-1 and JZ2-2-2 (6:4)	-	-
JZ1-1-1 and JZ2-2-2 (7:3)	-	-
JZ1-1-1 and JZ2-2-2 (8:2)	3.89 ± 0.07 c	44.95
JZ1-1-1 and JZ2-2-2 (9:1)	-	-

Note: The above values represent the mean ± standard error. Different lowercase letters in the same column indicate composite bacterial suspensions treated at different ratios, and “-” indicates no inhibitory activity.

**Table 4:** Effect of composite bacterial suspension on the control efficacy and quality of *Colletotrichum coccodes* in potatoes (11d).

Treatment Group	Lesions Diameter (cm)	Control Effect (%)	Weight Loss (%)
CK	0.98 ± 0.02 e	-	5.85
T	3.98 ± 0.15 a	-	22.58
X	1.64 ± 0.05 c	71.34	8.24
Y	1.31 ± 0.06 d	81.40	7.01
Z	2.21 ± 0.03 b	44.47	15.45
M	1.12 ± 0.04 e	87.19	6.75

Note: The above values represent the mean ± standard error. Different lowercase letters in the same column indicate the size of the lesion diameter under different treatments. “-” indicates no inhibitory activity.

**Table 5:** Specificity testing of the probe.

No.	Strain	Ct	GenBank Acc. No.
1	*Pantoea agglomerans* JZ1-1-1	29.85	PQ849068
2	Potato tuber	nd	-
3	JZ1-1-1 and potato tuber + potato tuber	26.60	-
4	*Bacillus subtilis* JZ2-2-2	28.74	PQ849069
5	JZ2-2-2 and potato tuber + potato tuber	25.72	-
6	*Bacillus amyloliquefaciens* QS10-6	nd	PQ856826
7	*Bacillus atrophaeus* QS2-5	nd	PQ856824
8	*Bacillus velezensis* QS2-13	nd	PQ856825
9	*Bacillus safensis* BM-7	nd	P0835495
10	*Bacillus atrophaeus* QB-2-7	nd	PQ835496

Note: “nd” indicates that the Ct value was not detected, and “-” indicates that there is no gene accession number.

## Data Availability

Data are contained within the article.
